# Development of a Thermomechanical Treatment Mode for Stainless-Steel Rings

**DOI:** 10.3390/ma15144930

**Published:** 2022-07-15

**Authors:** Irina Volokitina, Ekaterina Siziakova, Roman Fediuk, Alexandr Kolesnikov

**Affiliations:** 1 Department of Metallurgy and Mining, Rudny Industrial Institute, Rudny 111500, Kazakhstan; irinka.vav@mail.ru; 2 Mineral Raw Material Processing Faculty, Saint Petersburg Mining University, 199106 St. Petersburg, Russia; 3 Polytechnic Institute, Far Eastern Federal University, 690922 Vladivostok, Russia; 4 Peter the Great St. Petersburg Polytechnic University, 195251 St. Petersburg, Russia; 5 Department of “Life Safety and Environmental Protection” M. Auezov, South Kazakhstan University, Shymkent 160012, Kazakhstan

**Keywords:** severe plastic deformation, stainless steel, thermomechanical treatment, microstructure, mechanical properties

## Abstract

This article describes a technology for the thermomechanical treatment of stainless-steel piston rings. This technology makes it possible to obtain rings with an optimal combination of plastic and strength properties that is essential for piston rings. The following thermomechanical treatment is suggested for piston rings manufacturing: quenching at 1050 °C, holding for 30 min and cooling in water, then straining by the HPT method for eight cycles at cryogenic temperature and annealing at a temperature up to 600 °C. The resulting microstructure consisted of fine austenite grains sized 0.3 μm and evenly distributed carbide particles. Annealing above this temperature led to the formation of ferrite in the structure; however, preserving the maximum fraction of austenitic component is very important, since the reduction of austenite in the structure will cause a deterioration of corrosion resistance. The strength properties of steel after such treatment increased by almost two times compared with the initial ones: microhardness increased from 980 MPa to 2425 MPa, relative elongation increased by 20%. The proposed technology will improve the strength and performance characteristics of piston rings, as well as increase their service life, which will lead to significant savings in the cost of repair, replacement and downtime.

## 1. Introduction

The drive for the highest possible efficiency in manufacturing is reflected in the growing development of complex production processes and specialized materials [1,2,3,4,5,6,7,8,9,10]. In terms of manufacturing process development, this means a tendency to reduce the number of different production steps while using materials as efficiently as possible [11,12,13,14,15]. In terms of material performance, against an increasingly important, lightweight design, high strength with good ductility is of great importance [16,17]. Most manufacturing processes for processing metallic materials cause local or global changes in material properties. These production properties, meanwhile, can be used purposefully to increase component performance or reduce material usage in reverse order. Therefore, synergistic effects can be used to improve overall part production efficiency by adapting the production process to purposefully adjust certain local material properties [18,19].

The elastic properties of piston rings can be prolonged, and their failure reduced to a minimum by selecting the correct alloy grade and method of thermo-mechanical treatment. As a consequence, combustion engine manufacturers around the world are constantly searching for new technologies for piston rings production. Thus, improving the strength and performance characteristics of piston rings is an important technical task.

Conventional ultrafine-grained materials with grain sizes in the scale of several micrometers are usually manufactured using thermomechanical processes. An ultrafine-grained structure cannot be obtained by classical methods because of dynamic reduction and recrystallization processes, as well as limitations in formability [20,21,22,23,24,25]. Ultra-thin and nanocrystalline materials have been the subject of extensive research for a long time because their mechanical properties allow us to expect great potential as structural materials. Such materials can be made using two opposing approaches [26,27,28,29]. The first approach, often referred to as the “bottom-up process”, is based on the aggregation of individual atoms or nanoscale particles to create a compact material. These include vapor deposition processes, electrolytic deposition processes and powder metallurgy methods [30,31,32]. These methods allow the production of nanocrystalline materials with very small grain sizes but with limited workpiece dimensions. They are not commonly used to make superfine cloth. The second approach, called the “top-down” process, is based on grinding the grains using severe plastic deformation (SPD). The SPD processes create ultra-thin cloths by applying extreme stretching at high hydrostatic pressures and low homologous temperatures [33,34,35,36,37]. The achievable minimum grain sizes depend on the material properties, but as a rule, grain sizes up to the nanocrystalline state are not achieved. The most used and investigated methods of severe plastic deformation are equal -channel angular pressing, multiple isothermal forging and high-pressure torsion.

Shear strain-based equal-channel angular pressing (ECAP) was first presented by Segal et al. [38] and is one of the most frequently used SPD methods. Over the past two decades, work related to ECAP of metallic materials has attracted considerable interest among scientists in both fields of metal physics and materials science. This interest arose because of the possibility to process large volumes [39,40,41]. One of the main purposes of such works is to grind metal grains to an ultrafine grained or nanostructured state. This ensures that such metals achieve a unique set of physical and mechanical properties [42,43,44]. The next objective of such studies is to investigate the mechanisms of formation of ultrafine grained structures in the SPD process, since ECAP can achieve very high degrees of strain. In this case, the shape and dimensions of the strained workpieces do not change. In recent works [45,46,47], ECAP was combined with cryogenic temperature, producing even better results.

In high-pressure torsion (HPT), shear strains are introduced into flat cylindrical specimens by torsion at high hydrostatic pressure stresses [48,49]. A number of different works [50,51] has found that SPD by the HPT method affects the structure of the material and increases the density of crystal lattice defects. Many scientists have succeeded in obtaining the smallest grain size microstructure in various materials using the HPT method rather than other SPD methods. As a result, it was possible to study the peculiarities of such a structure and evaluate its mechanical and physical properties. Material deformed by the HPT method is processed non-uniformly along the grains’ radius. As a result, grains far from the center are more deformed than those in the center of the disk, so the microstructure becomes anisotropic. Since in our case the ring workpiece will be strained, this disadvantage can be avoided.

It should also be noted that when a certain accumulated degree of deformation is reached, the grinding process slows down and then stops altogether. This phenomenon still does not have an accepted explanation. However, one of the possible reasons is that there is some equilibrium between the strain grinding of the grains and their thermo-activated growth [52,53]. Therefore, grinding the structure to a nanocrystalline level by SPD only is not yet possible in most cases. As a result, new technologies are needed to continue the process of grinding the microstructure down to the nanocrystalline state.

There are several papers [54,55,56] that demonstrate the activation of new strain mechanisms by low temperatures. Such mechanisms suggest the possibility of continuing the process of grain refinement and reaching the nanoscale level. Lowering the temperature to cryogenic values provides improved mechanical properties and, as a result, increased wear resistance and hardness. The surface quality is also improved for polishing or finishing, which is necessary for piston rings (the presence of soft and ductile austenitic areas in the surface layer structure prevents the creation of a homogeneous mirror surface).

The main purpose of this article was to develop a new combined processing technology for piston rings used in internal combustion engines which will improve their performance and mechanical properties. This new technology combines high-pressure torsion with cryogenic temperature.

## 2. Materials and Methods

A special construction was developed to implement the HPT process on the existing equipment of the laboratory through the rectilinear movement of the upper die relative to the bed. The rectilinear motion of the upper die with the lower die attached to it transmits a torque due to the contact friction forcing directed at an oblique angle to the response part of the die. As a result, the rectilinear movement turns into a torsional movement.

**Figure 1:**
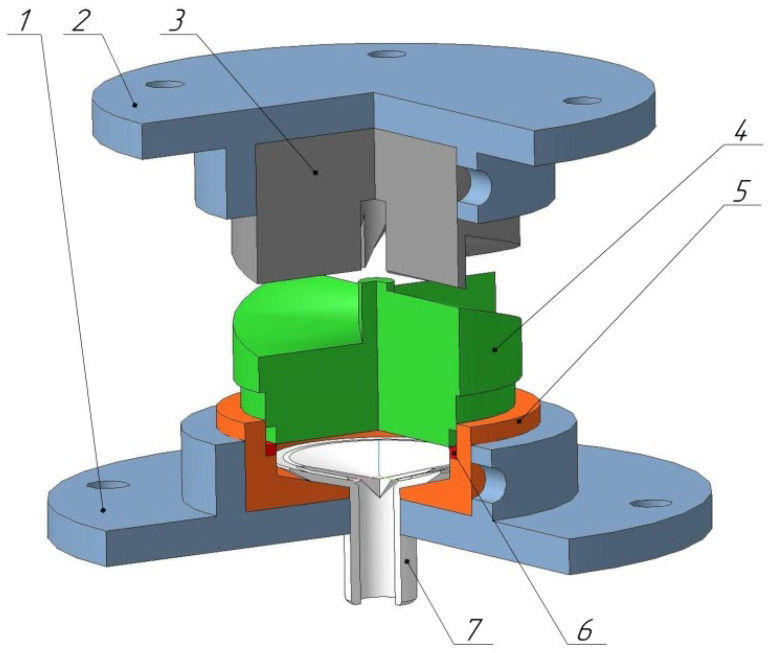
General view of the complete construction: 1—bottom carrier, 2—top carrier, 3—upper striker, 4—lower striker, 5—matrix, 6—piston ring, 7—nozzle.

Drawings were developed based on the analysis of scientific and technical literature and modeling in the Deform program package. The construction consists of several parts: an upper die which receives progressive motion from the press; a lower die which receives torque from the progressive motion of the upper die and the matrix itself, which holds the workpiece in the form of a ring (Figure 1).

Four spiral notches are present on the lower edge of the upper die. There is a cylindrical hole in the center of the upper die for the strain gauge rod and to ensure the alignment of the two dies.

The lower die has three steps. This structural solution is required for the straining of a ring workpiece (as in this case, rather than of a disk workpiece). The first pass (the second intermediate step) provides a contact with the side edge of the die where the workpiece is inserted. The second pass (the third lower step) provides contact with the workpiece along its inner radius, completely closing its cross section. According to this principle, the inner form of the matrix should also have a stepped form. The width of the step should correspond to the width of the circular workpiece to be machined.

A technological hole was made in the lower die to implement the high-pressure torsion process at cryogenic temperature. This hole was used to supply liquid nitrogen into the workpiece strain chamber. During the modeling, it was decided to use sprinkler-type nozzles, as, contrary to conventional nozzles, they allowed a uniform supply of nitrogen to the entire ring surface. These nozzles were made from polyurethane using a 3D printer.

**Figure 2:**
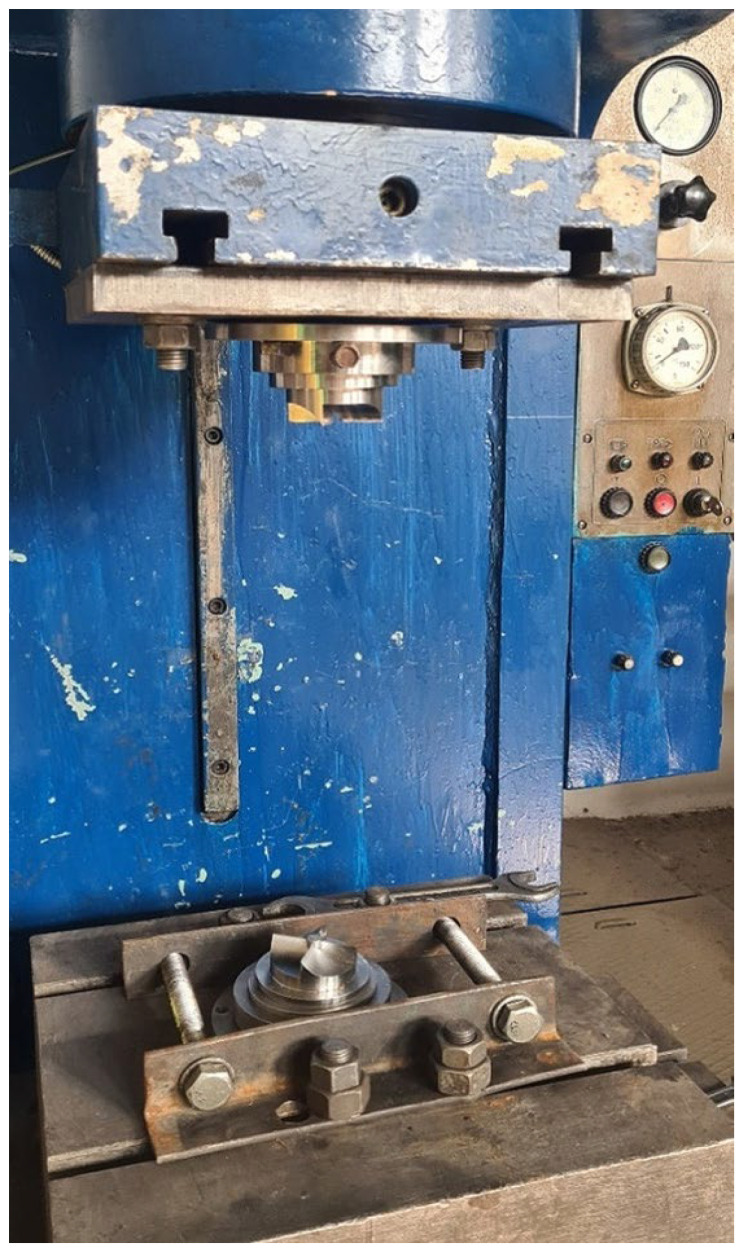
Construction fixed on the press.

The experiment itself and the assembly of the structure were carried out in the University laboratory on a single-curve hot-stamping press, model PB 6330-02, whose force was 1000 kN (Figure 2). Since there a martensitic transformation occurs in austenitic steels, the strain was applied out at cryogenic temperature and at room temperature for comparison. Therefore, depending on the temperature at which the workpieces were strained, the amount of martensite in the structure could vary greatly. The number of strain cycles was 8.

The deformation blanks were ring-shaped, 76 mm in diameter, 3.5 mm wide and 3 mm in thickness. Since the piston rings do not work in aggressive media, AISI-304 austenitic stainless steel was chosen as the workpiece material. The initial structure before strain was obtained by quenching at 1050 °C, holding at this temperature for 30 min, and cooling in water. After such preheating, the γ-solid solution was fixed in chromium–nickel steel.

It is known that a significant disadvantage of strongly deformed materials is their very low ductility which limits the possibility of their practical application. The plastic properties of such material can be recovered by applying a final heat treatment. Therefore, a laboratory experiment was conducted to increase the ductility of the deformed samples obtained with the HPT method. The samples were cut into thin plates with a thickness of 5 mm after HPT and subjected to holding at temperatures of 300–650 °C for 15 min, followed by cooling in water.

The structure was studied using a JEM2100 transmission electron microscope (TEM) (Akishima, Japan) with a magnification range of 1000 to 50,000 times. Thin foil for the microstructure study was prepared by thinning using electrolytic polishing in an electrolyte consisting of 400 mL of H_3_PO_4_ and 60 g of CrO_3_ at room temperature and voltage of 20 V, with current density of 2.5 A/cm^2^. For a more objective interpretation of the structure and the analysis of transformations, an a EBSD analysis was carried out using Philips XL-30 SEM (Amsterdam, The Netherlands) with a field cathode at an accelerating voltage of 20 kV. The results were processed using the Tex SEM Lab software 4.2. The scans were performed on 50 μm × 50 μm sections at 0.2 μm increments. Given the experimental accuracy of the EBSD method, all low-angle boundaries with a disorientation of less than 2° were excluded from consideration.

The microhardness of the samples was measured by the Vickers method using a DM-8 automatic microhardness tester (Affri, Induno, Italy). The load was 1 N.

Mechanical uniaxial tension tests were performed at room temperature on an Instron 5882 machine at a deformation rate of 1.0 mm/min. The sample deformation was measured with an Instron strain gauge. Tensile tests were carried out on flat samples cut from the ring (working part dimensions: width of 3 mm, thickness of 3 mm and length of 6 mm) in accordance with GOST 1497-84 recommendations. Tensile tests of mechanical properties were carried out to determine strength and ductility characteristics: yield strength (σ_0.2_), tensile strength (σ_B_) and maximum elongation to failure (δ).

## 3. Results

**Figure 3:**
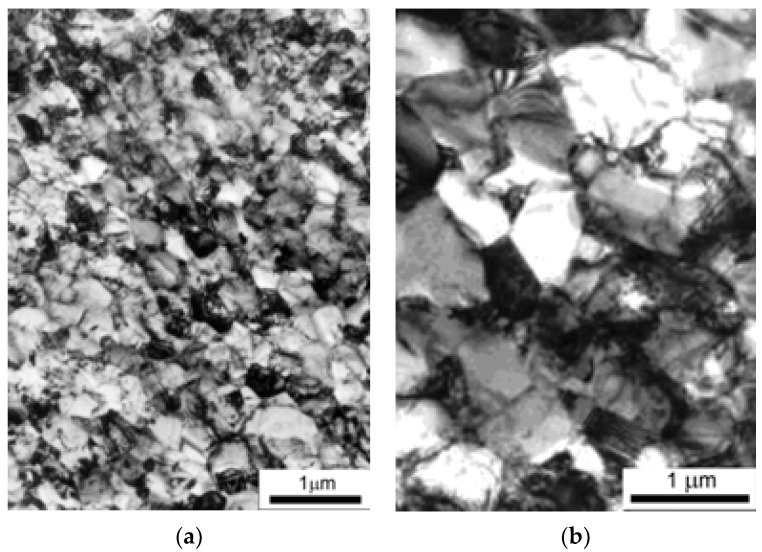
Microstructure of AISI-304 steel after straining by HPT: (**a**) at cryogenic temperature; (**b**) at room temperature.

The microstructure of AISI-304 stainless steel prior to HPT was coarse-grained with polyhedral grains, with an average size of 32 μm and the presence of twins. The structure contained ≈100% austenite (after a preliminary heat treatment—quenching). The microstructure obtained after applying the strain by the HPT method at room temperature and using cryogenic temperature is shown in Figure 3.

**Figure 4:**
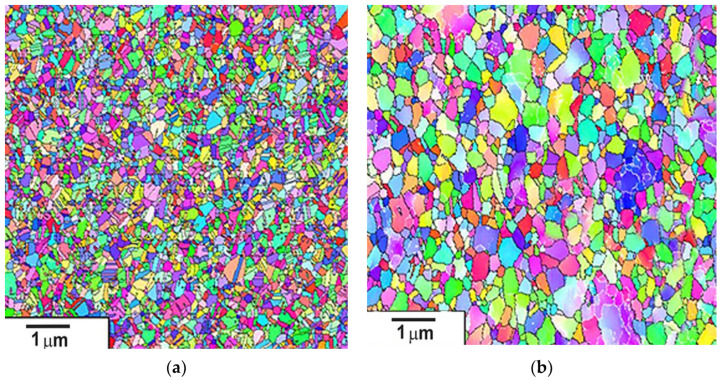
Microstructure orientation maps of AISI-304 steel after straining by HPT: (**a**) at cryogenic temperature; (**b**) at room temperature.

EBSD analysis was performed to obtain additional information on grain size, texture and disorientation of the boundaries. Orientation maps of the microstructure of AISI-304 steel after eight cycles of high-pressure torsion deformation are shown in Figure 4.

**Figure 5:**
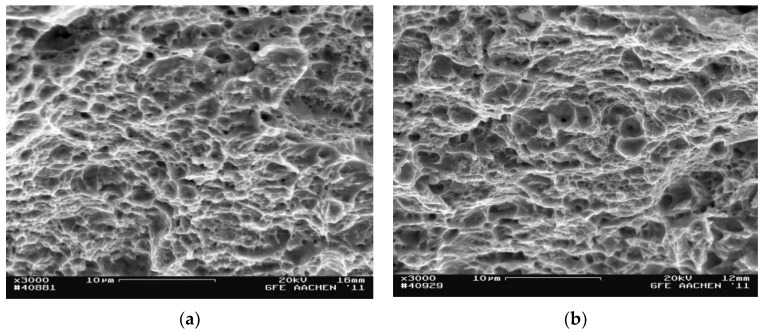
Fractography of the fracture surface in the crack start zone: (**a**) at cryogenic temperature; (**b**) at room temperature.

**Figure 6:**
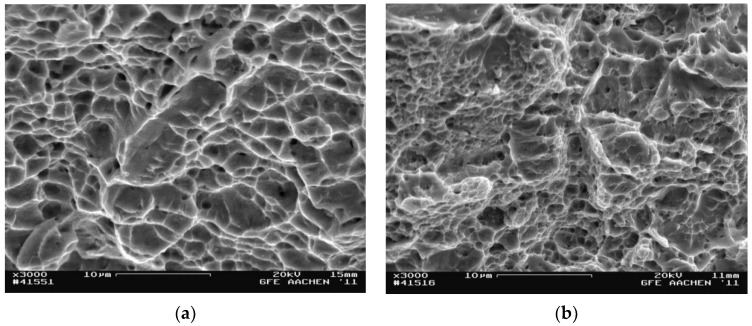
Fractography of the fracture surface in the crack growth zone: (**a**) at cryogenic temperature; (**b**) at room temperature.

Tensile tests were carried out to determine the mechanical characteristics after metallographic studies. In addition, interrupted tensile tests were performed to record the progress of the start zone and the crack growth zone as fully as possible. Tests on the samples obtained after applying the strain by the HPT method at room temperature were carried out at room and at cryogenic temperature. Photographs of the samples’ surface fractography shown in Figure 5 and Figure 6 were analyzed using SEM. Each photo shows the average area of the fracture at 3000× magnification.

**Figure 7:**
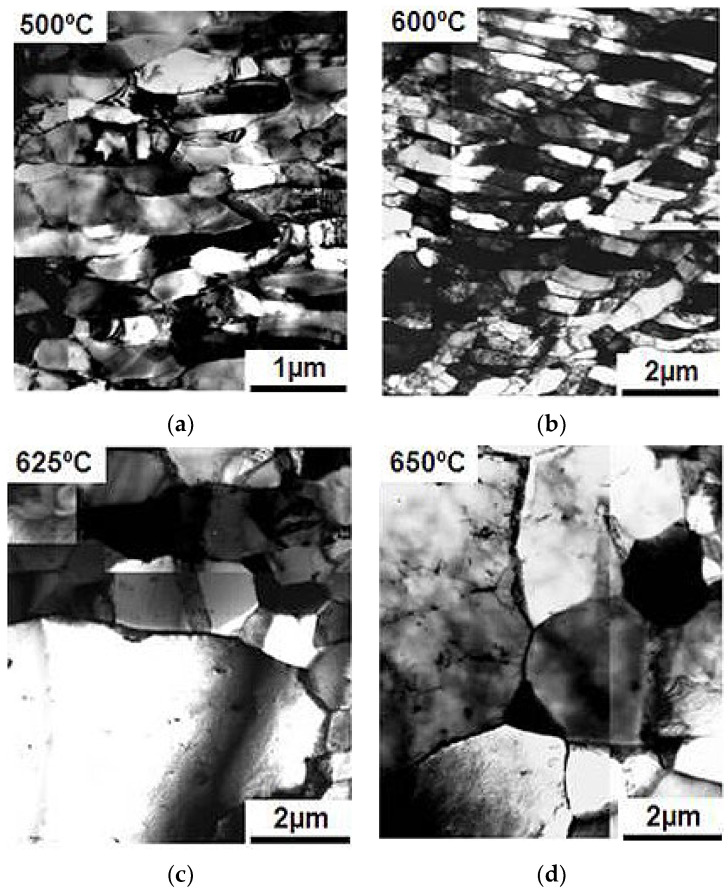
Microstructure of steel deformed with the HPT method at cryogenic temperature during heating: (**a**) 500 °C; (**b**) 600 °C; (**c**) 625 °C; (**d**) 650 °C.

**Figure 8:**
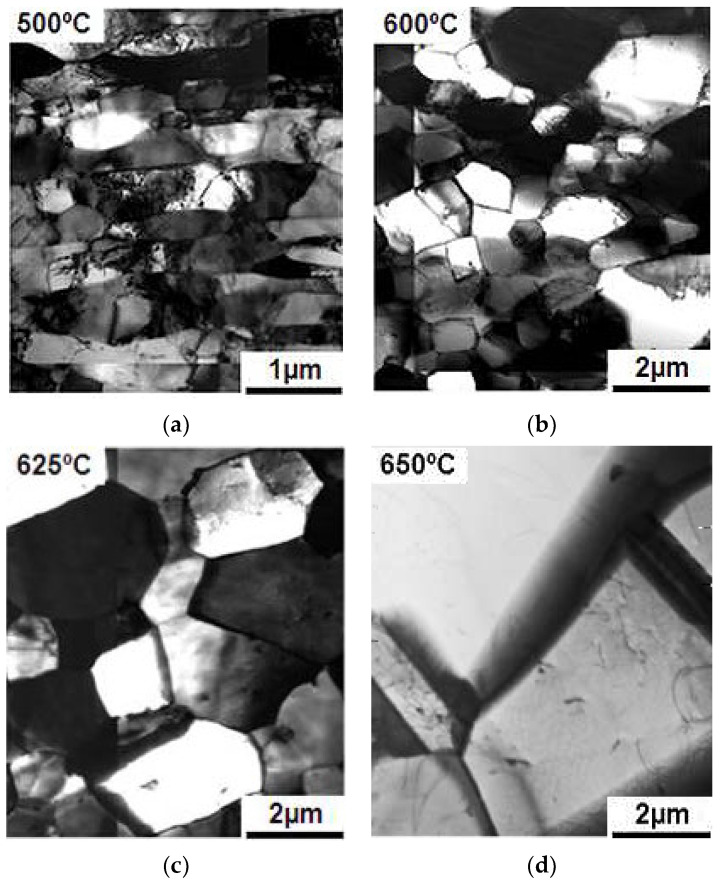
Microstructure of steel obtained with the HPT method at room temperature when heated at (**a**) 500 °C; (**b**) 600 °C; (**c**) 625 °C; (**d**) 650 °C.

Samples after deformation through the HPT method were annealed at temperatures of 300–650 °C with an exposure time of 15 min to observe changes in the microstructure. This was done to check the possibility of preserving the mechanical properties and microstructure during heating. The evolution of the microstructure of the deformed samples during heating is shown in Figure 7 and Figure 8.

**Figure 9:**
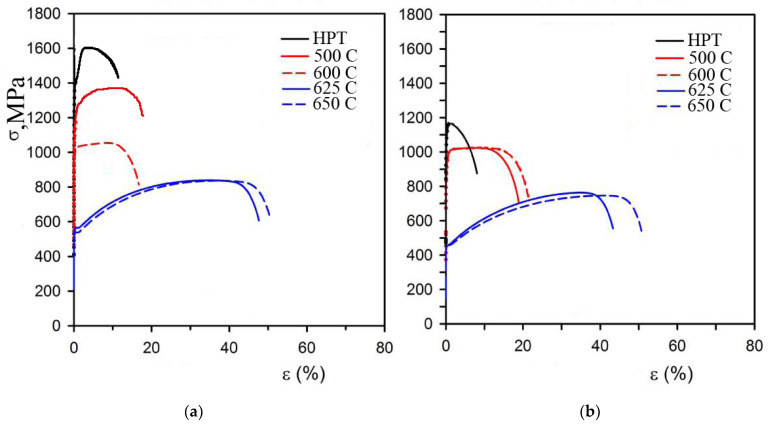
Tensile diagrams of samples obtained after HPT and heating during deformation: (**a**) at cryogenic temperature; (**b**) at room temperature.

After the strain and heating tests, mechanical tensile tests were carried out. The diagrams are shown in Figure 9.

## 4. Discussion

Analysis of the microstructure of the samples after deformation by the HPT method at room and cryogenic temperatures showed that eight strain cycles resulted in a homogeneous nanostructure in both cases (Figure 3). However, using cryogenic temperature resulted in a finer-grained structure. The cryogenic temperature resulted in a martensitic structure with a grain size of 0.2 μm (Figure 3a), whereas the room temperature resulted in a 0.5 μm microstructure consisting of a mixture of austenite and α-martensite (Figure 3b).

The EBSD analysis showed that the misorientation of the strain sub-boundaries increased during the deformation at both temperatures, i.e., the fraction of low-angle sub-boundaries decreased with increasing true strain. Strain twinning, which is characteristic at low strain rates, decreased after eight deformation cycles at room temperature, which was confirmed by the elimination of the peak with a misorientation angle of ~60. Intensive twinning was also observed at the eighth strain cycle when using cryogenic temperature. As a result, after eight deformation cycles with intensive cooling, the structure contained a large number of twins ~57%, while at room temperature, only 13% of twins were observed.

The distribution of boundaries on the angles of misorientation in both states was close. With conventional HPT, the fraction of low-angle boundaries was 18%, while with nitrogen treatment, it was 12%. The results showed that the total proportion of large-angle boundaries was at least 80%. This indicates the formation of a nanostructure with a predominance of large-angle boundaries in the workpieces.

The tensile tests showed that the obtained nanocrystalline structure had in both cases increased strength properties (Figure 9). In the initial state, we determined a yield strength of—275 MPa, a tensile strength of 515 MPa, and relative elongation of 40%. The formation of a nanocrystalline structure after eight cycles of strain by the HPT method at cryogenic temperature with a grain size of 45–50 nm led to an increase in the tensile strength to 1603 MPa compared to the initial state. The yield strength increased to 1282 MPa. The value of ductility decreased sharply up to 18% compared to the initial state. The samples deformed at room temperature showed the following mechanical properties: the tensile strength increased to 1198 MPa, and the yield strength increased to 1005 MPa. The value of ductility decreased up to 9% compared to the initial state.

The microhardness results correlated with the mechanical tensile test data and indicated that high-pressure torsion in the new die allowed obtaining a fairly homogeneous hardness across the entire cross section of the ring. After eight HPT cycles at cryogenic temperature, the microhardness increased almost three times compared to the initial state: from 980 MPa to 2715 MPa. Strain at room temperature resulted in an increase in microhardness to 2530 MPa. In this case, the main increase in hardness was in the first four passes—40%.

Low plastic properties were obtained based on the results of the study of mechanical properties using both deformation methods. This is a significant disadvantage of almost all samples obtained by severe plastic deformation methods [57,58,59]. Therefore, it is necessary to carry out additional research on crack growth during fracture.

The fracture surface of AISI 304 steel strained at cryogenic temperature in the crack start zone had a quasi-viscous nature. This was characterized by a ductile fracture mechanism. This was confirmed, as shown in Figure 5a, by the presence of spalling that alternated with dimpled fracture. The samples strained at room temperature had a brittle–ductile fracture in the crack start zone (Figure 5b).

The crack in the growth zone in the steel deformed at cryogenic temperature formed according to a microviscosity mechanism, and the pits were evenly spaced along the fracture surface (Figure 6a). The fracture surface in samples strained at room temperature was covered with spalls and pits indicating that the crack was spreading in a quasi-brittle way.

Based on the data obtained, cryogenic temperature allowed better plastic properties due to the martensite structure obtained in the samples, but these properties were still insufficient for further use. To increase the plastic properties, it is necessary to reduce the internal stress; this could be achieved by annealing. Annealing to 500 °C did not change the microstructure of samples processed at both cryogenic and room temperatures, and only a rearrangement of dislocations occurred (Figure 7a and Figure 8a). Recrystallization began at 600 °C in the samples obtained by deformation at room temperature. This was shown by the separately occurring grains, while no annealing twins were observed yet (Figure 8c). There were still no changes in the microstructure in samples processed at cryogenic temperature (Figure 7c). When the samples obtained with room temperature deformation reached 625 °C, a completely recrystallized structure with an average grain size of 2 μm was observed, and annealing twins were observed (Figure 8c). The samples processed at cryogenic temperature started recrystallization at 625 °C which caused a strong growth of individual grains above 3 μm (Figure 7c). There were also annealing twins. Once the structure reached 650 °C, it became completely recrystallized with a grain size of 3 μm (Figure 7d). The effects of annealing at 650 °C on samples strained at room temperature did not practically differ from those on samples obtained at cryogenic temperature. The structure was fully recrystallized, with a grain size of 4 μm (Figure 8d).

The rate of grain growth in HPT-processed samples at cryogenic temperature after heating was several times faster than in samples HPT strained at room temperature. This can be explained by a more intense deformation occurring at cryogenic temperature and a correspondingly higher degree of recrystallization.

Another feature of the structural change during steel heating was the development of a reverse-phase transformation of strained martensite back to austenite or ferrite.

The thermal stability of steel after HPT was also studied by analyzing the dependence of its microhardness change on the annealing temperature. We observed a slight decrease in microhardness when heating specimens deformed at both cryogenic and room temperature. The decrease was greater as the is the heating temperature increased, but it remained at a level much higher than that of microhardness after hardening. Therefore, during annealing up to 500 °C, the microhardness of samples which had been deformed at cryogenic temperature decreased from 2715 to 2555 MPa, and that of samples deformed at room temperature decreased from 2530 to 2205 MPa.

At 600 °C, the samples obtained by deformation at room temperature showed a sharp decrease in microhardness, which reached 1365 MPa. This indicated the beginning of recrystallization processes. After annealing at 650 °C, microhardness decreased to 985 MPa.

The same was observed in the samples deformed at cryogenic temperature: when heating to 625 °C, we observed a sharp decrease in microhardness from 2555 to 1725 MPa; when annealing at 650 °C, microhardness was 1260 MPa.

## 5. Conclusions

The results showed that during high-pressure torsion at cryogenic and room temperatures during the first strain cycles, the difference in grain size and structure was not large, since the adiabatic effects were comparable. Cryogenic temperature became effective only after four cycles of deformation, when the defect density increased dramatically. The structure of samples strained at both temperatures was refined to a nanostructure level. Thus, straining AISI-316 steel with an initial grain size of 32 μm at room temperature led to an equiaxed homogeneous microstructure of 0.5 μm. The structure consisted of a mixture of austenite and α-martensite. Straining at cryogenic temperature resulted in an equiaxial homogeneous microstructure sized 0.2 μm, consisting of 90% α-martensite with a predominance of large-angle boundaries.

Austenitic steel after annealing at 600 °C showed an optimal combination of ductility and strength, which is essential for piston rings. Therefore, the following thermomechanical treatment is proposed for piston rings manufacturing: quenching at 1050 °C, holding for 30 min and cooling in water, then straining by the HPT method for eight cycles at cryogenic temperature and annealing at a temperature up to 600 °C. As a result of this treatment, the microstructure will consist of fine austenite grains sized 0.3 μm and evenly distributed carbide particles.

## Data Availability

Data sharing is not applicable to this article.
